# Simultaneous Determination of Five Chromones of Radix *Saposhnikoviae* Extract in Rat Plasma by UPLC-MS/MS: Application to a Comparative Pharmacokinetic Study in Normal and Febrile Rats

**DOI:** 10.1155/2019/6454252

**Published:** 2019-02-26

**Authors:** Li Meng, Hui Gao, Bin Chen, Peng-peng Liu, Guo-shun Shan, Fan Zhang, Tian-zhu Jia

**Affiliations:** School of Pharmacy, Liaoning University of Traditional Chinese Medicine, Dalian 116600, China

## Abstract

A rapid and sensitive quantitative analytical method was established for the simultaneous determination of five chromones (prim-O-glucosylcimifugin, cimifugin, 4′-O-*β*-D-glucosyl-5-O-methylvisamminol, 5-O-methylvisammiol, and sec-o-glucosylhamaudol) in the plasma of RS-treated rats for the first time using ultra performance liquid chromatography- (UPLC-) tandem mass spectrometry. The Waters Acquity UPLC BEH C18 (50 mm × 2.1 mm, 1.7 *μ*m) was used as the chromatographic column, 0.1% formic acid water and 0.1% formic acid acetonitrile comprised the mobile phases, and all samples were determined under positive ion mode. The results showed that all analytes had good linearity (*r* > 0.9902), between-day and within-day precisions less than 15%, accuracy between −5.50% and 5.53%, and extraction recovery between 88.26% and 97.65%. Both the matrix effect and stability met the requirements. This method was successfully applied for the comparative pharmacokinetics of five active components of RS in normal and febrile rats. The results showed that the pharmacokinetic behavior of RS extract significantly differed between the two types of rats.

## 1. Introduction

Fever is a common symptom in the progression of many diseases, especially infectious diseases. Excessive or persistent fever can cause physical exertion and can even be life-threatening in severe cases. An increase in body temperature is dependent on the pathological changes in the body; therefore, fever often signals the development and progression of diseases. While most antipyretic chemicals have rapid therapeutic effects, they also have shorter durations of action due to their shorter half-lives in the body; thus, repeated administration is sometimes necessary to maintain efficacy. In addition, chemical drug administration is often accompanied by side effects such as lethargy, mental retardation, and liver injury. By contrast, traditional Chinese medicines (TCMs) are not single substances, they have fewer side effects, and their oral administration allows slower *in vivo* metabolism and a longer duration of action. Therefore, they have certain advantages when used as antipyretic drugs.

The TCM Radix *Saposhnikoviae* (RS) is the dried root of *S. divaricate* (*Turcz.*) Schischk in the family *Umbelliferae*. It can expel pathogenic factors from the body's surface, remove dampness to relieve pain, and relieve convulsions; thus, it is clinically used to treat symptoms such as cold, fever, headache, and arthritis caused by dampness [1]. As a TCM, the single medicine and compound preparations of RS are widely used in the clinic, and about 8% of prescriptions in the Chinese Pharmacopoeia contain RS. In recent years, the pharmacological activity and chemical constituents of RS have been extensively studied by researchers worldwide. Pharmacological studies have shown that the ethanol extract of RS has anti-inflammatory [2–4], antioxidant [5–7], analgesic [8], antipyretic [9], anticancer [10], and antiviral effects [11]. RS mainly contains chromones [12], volatile oils, coumarins [13], polysaccharides [14], and organic acids, with chromones being the main active components. Because prim-O-glucosylcimifugin and 4′-O-*β*-D-glucosyl-5-O-methylvisamminol [15–18] are the two most abundant chromones in RS, the total amounts of these two compounds are used as the chemical index for evaluating the quality of RS in the *Chinese Pharmacopoeia*. Previous studies on the pharmacokinetics (PKs) of RS have been reported, but they have been limited to the PKs of prim-O-glucosylcimifugin and 4′-O-*β*-D-glucosyl-5-O-methylvisamminol [19, 20] or single active components such as prim-O-glucosylcimifugin and sec-o-glucosylhamaudol in normal rats [21, 22]. However, simultaneous measurements of the contents of prim-O-glucosylcimifugin, cimifugin, 4′-O-*β*-D-glucosyl-5-O-methylvisamminol, 5-O-methylvisammiol, and sec-o-glucosylhamaudol in plasma after the oral administration of RS and the comparison of PKs in normal rats and febrile animal models have not been reported.

Some studies have shown that the disease state can change the PK parameters of drugs [23]. The curative effects of some TCMs can only be reflected when the body is in a specific pathological state; therefore, metabolic studies of TCM components in only normal physiological conditions is not sufficient to fully evaluate the efficacy. Hence, studying the PK properties of drugs such as absorption, distribution, metabolism, and excretion in disease models can better explain the PK characteristics of drugs [24].

In this study, the levels of five types of chromones in the plasma of normal and febrile rats after oral administration of RS were determined using ultra performance liquid chromatography-tandem mass spectrometry (UPLC-MS/MS), and the PK characteristics of the active components in the plasma were compared. The results of this study provide a scientific basis for the antipyretic mechanisms of RS and its future clinical applications.

## 2. Materials and Methods

### 2.1. Chemicals and Reagents

**Figure 1:**
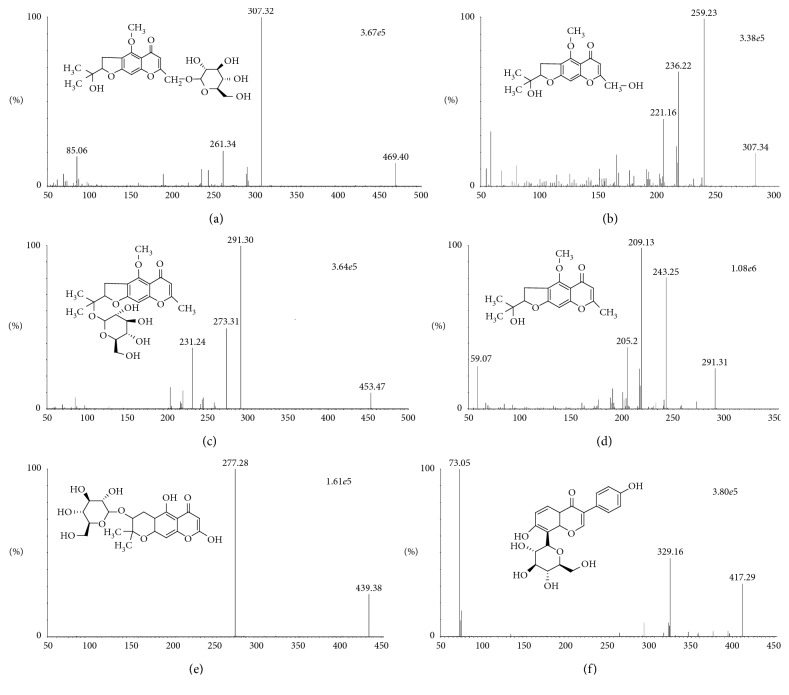
The chemical structures and full-scan product ion spectra of prim-O-glucosylcimifugin (a), cimifugin (b), 4′-O-*β*-D-glucosyl-5-O-methylvisamminol (c), 5-O-methylvisamminol (d), sec-O-glucosylhamaudol (e), and puerarin (IS) (f) in the positive ionization mode.

Standards for cimifugin and 4′-O-*β*-D-glucosyl-5-O-methylvisamminol were purchased from the National Institutes for Food and Drug Control (Beijing, China), and standards for prim-O-glucosylcimifugin, 5-O-methylvisammiol, sec-o-glucosylhamaudol, and puerarin were purchased from Chengdu Mansite Pharmaceutical Co., Ltd. (Sichuan, China). The purities of all standards and internal standard (IS) were higher than 98%, and their chemical structures are shown in Figure 1. RS was purchased from Hebei Anguo Medicine Market and was identified as the dried roots of *S. divaricate (Trucz.*) Schischk by Professor Feng Li from the College of Pharmacy, Liaoning University of Traditional Chinese Medicine (Liaoning Sheng, China). Acetonitrile (HPLC-grade) was obtained from Tedia Scientific (Fairlawn, NJ, USA). Pure water was supplied by Wahaha Company (Hangzhou, China). All other chemicals and solvents were of the highest analytical grade available.

### 2.2. Animals

Male-specific pathogen-free Sprague-Dawley rats with body weights between 180 and 220 g were purchased from Changsheng Bio-Technology (Certification No.: SCXK (Liao) 2010–0001; Liaoning, China). All rats were kept in a room maintained under a controlled 12 h light-dark cycle at a temperature of (24 ± 2)°C with 40–60% humidity and could drink water and eat *ad libitum*. All rats were housed for 7 days before the experiments were conducted. The body temperature of the rats was measured during feeding at 30 min intervals using a rectal thermometer, and rats with temperature fluctuations less than 0.5°C were selected for further experiments.

### 2.3. UPLC-MS/MS Conditions

The chromatographic column used was Waters Acquity BEH C18 (50 mm × 2.1 mm, 1.7 *μ*m), mobile phase A was 0.1% formic acid water, and mobile phase B was 0.1% formic acid acetonitrile. Gradient elution was performed as follows: 0–1.5 min, 10–15% mobile phase B; 1.5–3.5 min, 15–30% mobile phase B; 3.5–4.5 min, 30–40% mobile phase B; 4.5–6 min, 40–70% mobile phase B; 6–7 min, 70–10% mobile phase B; 7-8 min, 10% mobile phase B. The flow rate was 0.3 mL·min^−1^, the oven temperature was 35°C, and the injection volume was 2 *μ*l.

Mass spectrometry was conducted using the Waters Xevo TQD Triple Quadrupole equipped with an electrospray ionization source in the positive ion mode, 2 kV capillary voltage, 30 V cone voltage, ion source temperature of 250°C, desolvation gas temperature of 400°C, desolvation gas flow rate of 800 L/h, and cone gas flow of 50 L/h. Data were collected and analyzed using MassHunter Version 4.0 and DAS 2.0 software. The plasma samples were analyzed and quantified by multiple reaction monitoring (MRM), and the MRM parameters of each component are shown in Table 1.

### 2.4. Preparation of RS Extract

RS (150 g) was refluxly extracted twice for 2 h each in 10-fold volume of water. The extracts were filtered and concentrated to 90 mL, and then stored at 4°C before use.

### 2.5. Preparation of Standard Solution and Quality Control

The appropriate amounts of prim-O-glucosylcimifugin, cimifugin, 4′-O-*β*-D-glucosyl-5-O-methylvisamminol, 5-O-methylvisammiol, and sec-o-glucosylhamaudol standards were accurately weighed and dissolved in methanol as standard stocks. The mixed standard stock (prim-O-glucosylcimifugin 6.34 *μ*g/mL, cimifugin 26.44 *μ*g/mL, 4′-O-*β*-D-glucosyl-5-O-methylvisamminol 2.468 *μ*g/mL, 5-O-methylvisammiol 17.736 *μ*g/mL, and sec-o-glucosylhamaudol 1.104 *μ*g/mL) was made by accurately mixing the relative amount of standard stocks, and then diluting into a series of mixed standard samples using methanol. The concentration ranges of the standards were (prim-O-glucosylcimifugin) 0.008–3.170 *μ*g/mL, (cimifugin) 0.033–13.220 *μ*g/mL, (4′-O-*β*-D-glucosyl-5-O-methylvisamminol) 0.003–1.234 *μ*g/mL, (5-O-methylvisammiol) 0.011–4.434 *μ*g/mL, and (sec-o-glucosylhamaudol) 0.001–0.552 *μ*g/mL. Mixed standards with low, medium, and high concentrations for quality control (QC) were made using the same method, and the concentrations were 0.024, 0.237, and 2.378 *μ*g/mL for prim-O-glucosylcimifugin; 0.099, 0.992, and 9.915 *μ*g/mL for cimifugin; 0.009, 0.092, and 0.926 *μ*g/mL for 4′-O-*β*-D-glucosyl-5-O-methylvisamminol; 0.033, 0.333, and 3.326 *μ*g/mL for 5-O-methylvisammiol; and 0.004, 0.042, and 0.414 *μ*g/mL for sec-o-glucosylhamaudol. Puerarin standard (IS) was diluted to 25.48 *μ*g/mL with methanol. All samples were stored at −4°C until use.

### 2.6. Preparation of Plasma Sample

A 100 *μ*L aliquot of each plasma sample was mixed with 20 *μ*L IS (0.025 *μ*g/mL puerarin) and mixed by vortexing for 30 s, followed by the addition of 400 *μ*L acetonitrile, and mixing by vortexing for 3 min. Then, the sample was centrifuged at 13000 rpm·min^−1^ for 10 min, and the supernatant was dried with nitrogen gas in a 37°C water bath. The residue was resuspended in 200 *μ*L initial mobile phase (10% acetonitrile), vortexed for 1 min, and centrifuged at 13000 rpm·min^−1^ for 10 min. The resulting supernatant was used for analysis.

### 2.7. Method Validation

The specificity, standard curve, lower limit of quantitation (LLOQ), recovery, matrix effect, precision, accuracy, and stability of this method were investigated according to the guidance document by the U.S. Food and Drug Administration entitled *Bioanalytical Method Validation Guidance for Industry* [25].

#### 2.7.1. Specificity

To evaluate the specificity of the method, we compared chromatographic peaks of the blank plasma (a mixture of blank plasma from six rats), blank plasma supplemented with prim-O-glucosylcimifugin, cimifugin, 4′-O-*β*-D-glucosyl-5-O-methylvisamminol, 5-O-methylvisammiol, sec-o-glucosylhamaudol, and IS, and the plasma after oral administration of RS, to investigate the interference of endogenous substances in control plasma to the analytes.

#### 2.7.2. Linearity and LLOQ

The two-sample *t*-test was used for analysis of the blank plasma samples supplemented with a series of mixed standards. The standard curves were obtained by taking the mass concentration ratio of the analytes to the IS as the *x*-coordinate and the peak area ratio of the analytes to the IS as the *y*-coordinate. Then, linear regression was conducted using the weighted least squares method (weighted coefficient 1/*x*^2^). The LLOQ was calculated as the lowest concentration of the standard curve determined by the signal-to-noise ratio, which was generally higher than 5. The resulting accuracy was expected to be between 80% and 120% with a relative standard deviation (RSD) less than 20%.

#### 2.7.3. Precision and Accuracy

For QC, six samples each of low, medium, and high concentrations and the sample at the concentration of LLOQ were analyzed, and within-day (measured on the same day) and between-day (measured on three consecutive days) precisions and accuracies were measured according to the concentrations of samples. The precision was expressed as the RSD, which was expected to be less than 15%; and the accuracy was expressed as relative error (RE), which was expected to be within ± 15%.

#### 2.7.4. Recovery and Matrix Effect

The recovery refers to the peak area ratio of the QC samples to extracted blank plasma supplemented with standards and IS. The matrix effect is the peak area ratio of the IS in the extracted blank plasma supplemented with standards and IS and in the signal standards. The recovery and matrix effect of the sample at the concentration of LLOQ and a single concentration of puerarin (IS) were measured at the same time.

#### 2.7.5. Stability

The stability of the QC samples with low, medium, and high concentrations in different conditions such as room temperature, freezing, repeated freezing, and thawing were investigated including incubation at room temperature for 12 h, freezing at −80°C for 14 days, and three cycles of repeated freezing and thawing between −80°C and room temperature. Then, the samples were stored at 4°C for 12 h after treatment.

### 2.8. PK Study

Rats were randomly divided into two groups: the blank control group and the fever model group with six rats in each group. Rats in the fever model group were subcutaneously injected with 20% dry yeast suspension (10 mL·kg^−1^). After 4 h, all rats in both groups were intragastrically administered 20 mL/kg RS. Whole blood (0.5 mL) was taken from the orbital sinus after 0.083, 0.167, 0.333, 0.5, 1, 2, 4, 6, 8, 10, 12, and 24 h of administration. Blood samples were placed in anticoagulant tubes with heparin sodium and centrifuged at 3500 rpm·min^−1^, 4°C for 10 min to isolate the plasma. The plasma was stored at −80°C before use.

### 2.9. Statistical Analyses

Statistical analysis was conducted using SPSS 17.0 software, and the PK parameters were calculated using DAS 3.2 software (Chinese Pharmacological Society, Shanghai, China). The results and PK parameters are expressed as the mean ± standard deviation (SD). *P* values less than 0.05 were considered statistically significant.

## 3. Results and Discussion

### 3.1. Optimization of Sample Extraction

The pretreatment of biological samples is key to accurate determination. In this study, plasma was treated by either liquid-liquid extraction or protein precipitation. The liquid-liquid extraction method led to lower recovery and higher matrix effects of the plasma analytes, and was a more tedious procedure. Therefore, the protein precipitation method was used for the pretreatment of samples. Different precipitating reagents (ethyl acetate, methanol, and acetonitrile) were also compared, and acetonitrile led to better extraction of all analytes with no endogenous interference. Therefore, acetonitrile was used for protein precipitation for the pretreatment method of plasma samples.

### 3.2. Optimization of Chromatographic and Mass Spectra Conditions

To obtain better chromatographic results, the LC-MS analytical conditions were investigated prior to the experiments. The chromatographic behavior (peak symmetry and retention time) and mass spectra of the analytes are largely affected by the mobile phase. In this study, two mobile phase systems, water-methanol and water-acetonitrile, were investigated. The water-acetonitrile system led to higher analyte responses and lower background noise. The addition of low concentrations of formic acid to the mobile phase can improve the peak shape and sensitivity. Finally, the mobile phase was determined to be 0.1% formic acid water and 0.1% formic acid acetonitrile. All analytes were analyzed under both positive and negative ion modes, and the results showed that the positive ion mode had higher analyte responses, while the background noise was small; therefore, the positive ion mode was selected for detection. Based on these results, the capillary voltage, cone voltage, and collision energy were adjusted to further optimize the parameters of the analytes and the IS. The parameters of the analytes and the IS are shown in Table 2, and the full-scan ion spectrum and structure of the analytes and IS are shown in Figure 1.

### 3.3. Method Validation

#### 3.3.1. Specificity

**Figure 2:**
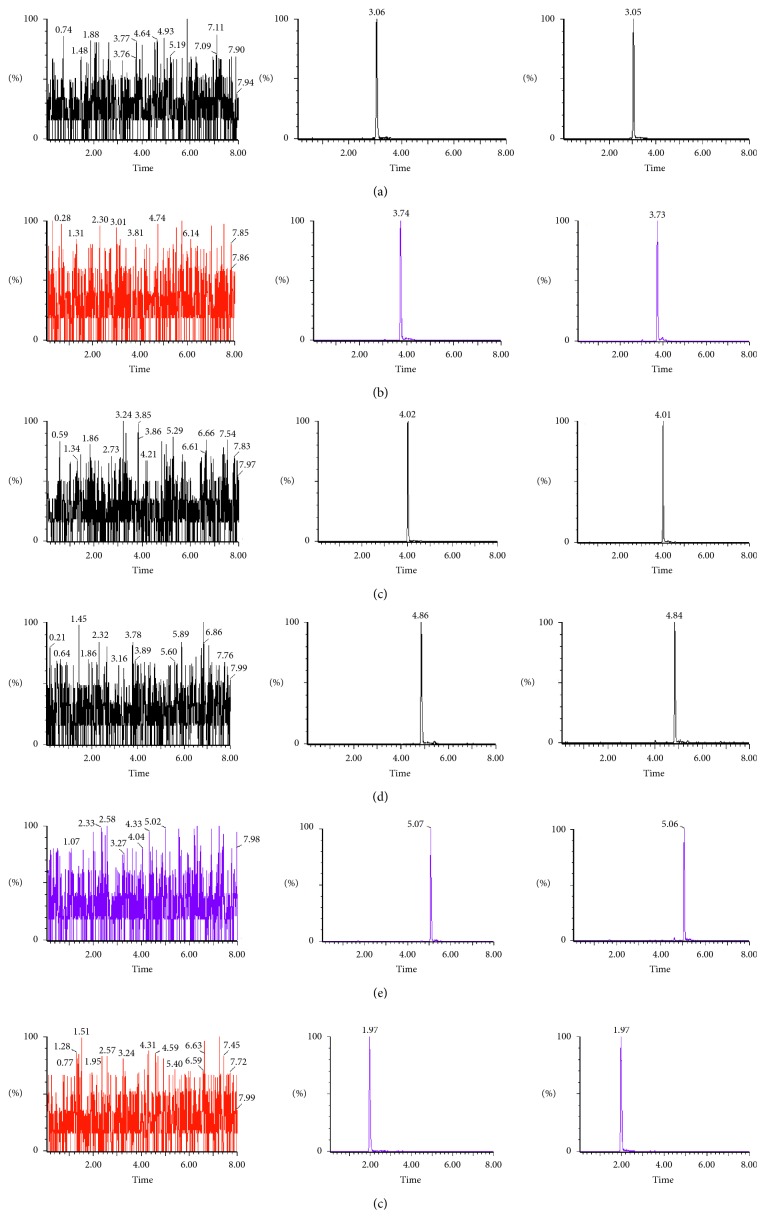
Representative MRM chromatograms of (a) prim-O-glucosylcimifugin, (b) cimifugin, (c) 4′-O-*β*-D-glucosyl-5-O-methylvisamminol, (d) 5-O-methylvisamminol, (e) sec-O-glucosylhamaudol, (f) IS in (I) blank plasma, (II) blank plasma spiked with the analytes at the LLOQ and IS, and (III) rat plasma sample obtained at 1 h after oral administration of RS extract.

As shown in Figure 2, under the selected experimental conditions, the indicator components had good resolution, and the retention times were 3.04, 3.73, 4.01, 4.84, and 5.05 min. The endogenous substances in the blank plasma had no interference with the determination of the indicator components, suggesting the good specificity of this method.

#### 3.3.2. Linearity and LLOQ

All indicator components had good linearity, with correlation coefficients higher than 0.9902. The LLOQ of prim-O-glucosylcimifugin, cimifugin, 4′-O-*β*-D-glucosyl-5-O-methylvisamminol, 5-O-methylvisammiol, and sec-o-glucosylhamaudol was 0.008, 0.033, 0.003, 0.011, and 0.001 *μ*g/mL, respectively. The results are shown in Table 2.

#### 3.3.3. Precision and Accuracy

The within-day and between-day precisions and accuracies of the high, medium, and low concentrations of QC samples are shown in Table 3. The between-day precisions of the QC samples ranged from 1.31% to 6.87%, and the accuracies ranged from −4.88% to 5.14%, whereas the within-day precisions ranged from 1.60% to 3.67%, and the accuracies ranged from −5.50% and 5.53%. All results met the analytical methodology requirements for biological samples.

#### 3.3.4. Recovery and Matrix Effect

The recoveries and matrix effects of the analytes and the IS are shown in Table 3. The recoveries of the high-, medium-, and low-concentration QC samples were between 88.26% and 97.65%, with an RSD < 6.69%, IS recovery of 91.21%, and RSD of 4.19%. The matrix effects of the high-, medium-, and low-concentration QC samples were between 86.50% and 102.85%, with an RSD < 6.58%, IS matrix effect of 101.88%, and RSD of 2.62%. All of these results were within an acceptable range and met the analytical methodology requirements for biological samples, which indicated that the extraction method ensured the accuracy, consistency, and repeatability of the experimental results and that the matrix effect had no effect on the analytical results.

#### 3.3.5. Stability

As shown in Table 4, the plasma samples had good stability during short-term storage, long-term cryopreservation, repeated freezing and thawing, and preservation after preparation, which showed no significant changes.

### 3.4. Pharmacokinetic Study

**Figure 3:**
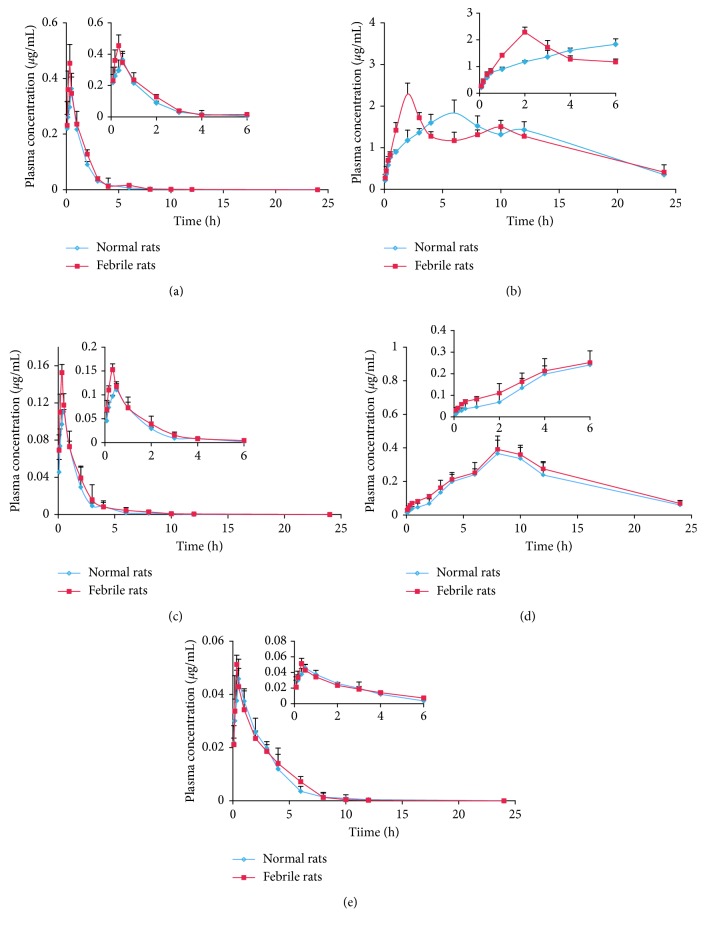
Mean plasma concentration-time curves for (a) prim-O-glucosylcimifugin, (b) cimifugin, (c) 4′-O-*β*-D-glucosyl-5-O-methylvisamminol, (d) 5-O-methylvisamminol, and (e) sec-O-glucosylhamaudol in normal and febrile rats after oral administration of RS extract, repectively (*n*=6).

**Figure 4:**
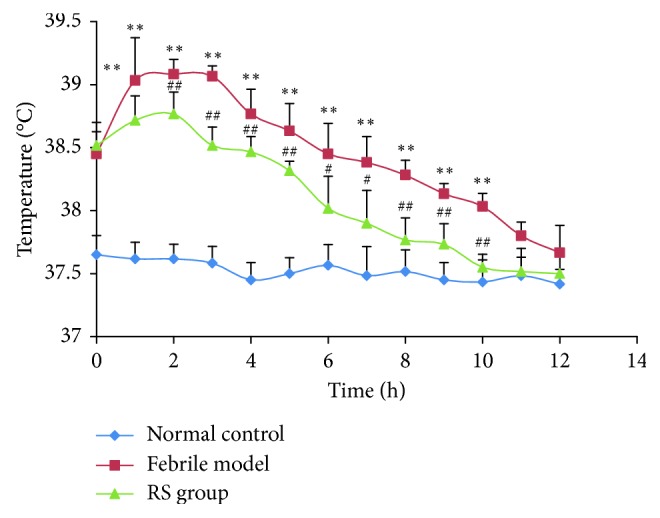
Antipyretic effect of RS in febrile rats. ^*∗∗*^*p* < 0.01, febrile model compared with normal control; ^#^*p* < 0.05, ^##^*p* < 0.01 RS group compared with febrile model. (mean ± SD, *n*=6).

An aliquot of 2 *μ*L treated plasma samples was injected into the LC mass spectrometer, and the PK data were analyzed using the noncompartment model. The main PK parameters are shown in Table 5, and the mean plasma concentration-time curves are shown in Figure 3. The results showed that the PK parameters of the RS extract in normal and febrile rats were significantly different. Compared with the control group, in febrile rats, plasma prim-O-glucosylcimifugin, cimifugin, and 4′-O-*β*-D-glucosyl-5-O-methylvisamminol had significantly increased *C*_max_ (*P* < 0.05) and significantly decreased *T*_max_ (*P* < 0.05), and plasma 4′-O-*β*-D-glucosyl had significantly increased AUC_0–*t*_ and AUC_0–∞_ (*P* < 0.05). There was no significant difference in the other parameters.  Figure 3(b) shows that the mean plasma concentration-time curves of cimifugin in both normal and febrile rats showed two peaks, consistent with previous studies [26–28]. These results suggest that cimifugin might be involved in the hepatoenteric circulation or that part of the extracted prim-O-glucosylcimifugin was enzymatically broken down to cimifugin, which was absorbed into the blood and led to the second cimifugin peak. Together with the PKs studies of RS, the effects of the RS extract on the body temperature of febrile rats were investigated. The body temperatures of the rats at different time points are shown in Figure 4. Compared with the control group, the body temperature of the febrile rat group significantly increased within 12 h of RS administration (*P* < 0.05), significantly decreased at 2 h after administration (*P* < 0.05), and then returned to normal at 8 h after administration. These results further confirmed the antipyretic effects of RS.

## 4. Conclusions

In this study, a quantitative analysis method for the simultaneous determination of five effective components in the plasma of rats after intragastric administration of RS extract was established for the first time using UPLC-MS/MS, and the PK parameters in normal and febrile rats were compared. This method had the advantages of a short detection time, good linearity, high recovery, and good stability, which met the requirements of analysis. The results of this study showed that, compared with the control group, the plasma concentration of prim-O-glucosylcimifugin, cimifugin, and 4′-O-*β*-D-glucosyl-5-O-methylvisamminol of the febrile rats had significantly increased *C*_max_ (*P* < 0.05) and significantly decreased *T*_max_ (*P* < 0.05), indicating that under conditions of high fever, these three compounds had higher absorption rates and blood intake volumes in rats. Therefore, the body changed the absorption pattern of the effective components in RS during fever, which provides a basis for future studies on the absorption patterns of the active components in RS. The mechanisms underlying how the febrile state influences PK behavior may be related to the changes of several mechanisms such as liver metabolism, renal excretion, and gastrointestinal transport [29–31]. Additional studies are needed to confirm this theory.

## Figures and Tables

**Table 1:** MS/MS transitions and parameters for detection of the analytes and internal standards.

Analytes	MRM	Cone voltage (V)	Collision energy (eV)
Prim-O-glucosylcimifugin	468.40 ⟶ 307.32	60	30
Cimifugin	307.34 ⟶ 259.23	55	20
4′-O-*β*-D-glucosyl-5-O-methylvisamminol	453.47 ⟶ 291.30	55	30
5-O-methylvisamminol	291.31 ⟶ 209.13	52	20
sec-O-glucosylhamaudol	439.38 ⟶ 277.28	28	16
Puerarin (IS)	417.29 ⟶ 73.05	32	28

**Table 2:** The regression equations and lower limit of quantification of the analytes.

Analytes	RT (min)	Calibration curves	*R*	Liner range (*μ*g/mL)	LLOQ (*μ*g/mL)
prim-O-glucosylcimifugin	3.04	*y* = 31.488*x* +* *0.117	0.9902	0.008–3.170	0.008
Cimifugin	3.73	*y* = 32.072*x* + 0.110	0.9950	0.033–13.220	0.033
4′-O-*β*-D-glucosyl-5-O-methylvisamminol	4.01	*y* = 49.868*x* + 0.039	0.9913	0.003–1.234	0.003
5-O-methylvisamminol	4.84	*y* = 161.731*x* + 0.062	0.9976	0.011–4.434	0.011
sec-O-glucosylhamaudol	5.06	*y* = 63.046*x* + 0.770	0.9957	0.001–0.552	0.001

**Table 3:** Precision, accuracy, extraction recovery, and matrix effect of the analytes (*n*=6).

Analytes	Intraday		Interday		Recovery		Matrix effect	
Concentration (*μ*g/mL)	Precision (RSD%)	Accuracy (RE%)	Precision (RSD%)	Accuracy (RE%)	Mean ± SD (%)	RSD (%)	Mean ± SD (%)	RSD (%)
Prim-O-glucosylcimifugin								
0.008	2.38	−1.18	1.46	−0.14	94.73 ± 4.41	4.65	93.25 ± 4.44	4.77
0.024	4.70	1.37	3.19	−5.50	93.35 ± 5.00	5.36	89.19 ± 4.45	4.98
0.238	2.91	−4.80	3.08	−2.81	93.03 ± 3.76	4.04	92.75 ± 1.91	2.06
2.378	2.89	−2.63	5.67	5.53	91.40 ± 4.35	4.76	90.35 ± 5.28	5.85

Cimifugin								
0.033	2.75	−0.45	2.67	−0.93	95.07 ± 3.90	4.10	96.93 ± 4.43	4.57
0.099	1.99	−2.68	3.03	0.69	90.72 ± 3.73	4.11	91.28 ± 2.93	3.20
0.992	2.51	2.32	3.22	−1.12	89.52 ± 4.73	5.29	86.50 ± 3.80	4.39
9.915	4.67	−1.69	5.31	−2.09	97.65 ± 2.89	2.75	98.37 ± 4.59	4.67

4′-O-*β*-D-glucosyl-5-O-methylvisamminol								
0.003	2.95	−2.59	4.13	−1.30	95.09 ± 4.98	5.23	98.30 ± 3.53	3.58
0.009	2.37	−4.10	2.57	2.47	93.66 ± 4.23	4.51	95.14 ± 3.96	4.16
0.093	1.31	−2.57	2.40	−1.04	88.75 ± 2.26	2.55	92.65 ± 1.96	2.12
0.926	6.87	5.14	1.60	−1.57	94.35 ± 4.66	4.94	89.75 ± 2.29	2.56

5-O-methylvisamminol								
0.011	4.20	2.98	2.83	2.37	94.37 ± 3.48	3.68	97.28 ± 3.46	3.56
0.033	5.33	−3.63	3.04	−4.25	91.15 ± 5.43	5.96	102.18 ± 3.86	3.78
0.333	4.23	4.33	2.82	−1.88	93.33 ± 5.44	5.82	89.30 ± 2.75	3.08
3.325	3.94	−3.4	3.02	0.35	95.87 ± 3.20	3.34	95.03 ± 4.84	5.09

Sec-O-glucosylhamaudol								
0.001	5.49	−2.78	5.13	−2.22	95.14 ± 4.47	4.70	98.23 ± 4.41	4.49
0.004	3.72	−4.88	3.58	1.46	93.05 ± 3.36	3.61	101.33 ± 2.97	2.93
0.041	2.82	−3.46	2.84	−1.15	97.03 ± 4.33	4.46	96.73 ± 6.37	6.58
0.414	3.18	1.04	3.57	−2.17	88.26 ± 5.91	6.69	102.85 ± 4.22	4.10
Puerarin (IS) 0.100					91.21 ± 5.82	4.19	101.88 ± 3.74	2.62

**Table 4:** The stability test of the analytes in rat plasma (*n*=6).

Analytes	At room temperature for 12 h		After three freeze-thaw cycles		At −80°C for 14 days		Post-treatment for 12 h at 4°C	
Concentration (*μ*g/mL)	RSD (%)	RE (%)	RSD (%)	RE (%)	RSD (%)	RE (%)	RSD (%)	RE (%)
Prim-O-glucosylcimifugin								
0.024	3.64	−3.20	7.40	3.70	2.13	−4.78	1.61	−3.97
0.238	3.06	−3.93	6.06	2.88	2.26	−5.83	1.49	−4.17
2.378	2.10	−1.38	1.86	−2.34	2.54	−1.49	4.36	2.51
Cimifugin								
0.099	3.72	1.46	4.75	−4.03	5.19	−3.84	3.29	−3.71
0.992	3.79	−1.86	4.35	−4.49	3.24	1.20	4.88	−1.66
9.915	4.24	−1.38	6.62	−2.26	3.22	1.06	3.74	−3.82

4′-O-*β*-D-glucosyl-5-O-methylvisamminol								
0.009	2.05	−4.07	2.36	−3.11	3.03	−4.22	1.16	0.86
0.093	1.86	4.17	2.68	−6.72	1.51	−5.96	4.34	−5.01
0.926	1.58	−2.29	2.04	2.47	2.87	−3.15	3.05	−2.64

5-O-methylvisamminol								
0.033	3.49	−2.78	4.41	−4.35	2.69	−3.34	2.83	1.36
0.333	2.96	4.94	3.93	−4.89	1.62	5.49	4.13	−3.00
3.326	3.73	−2.96	5.08	−3.13	2.59	2.17	3.48	−3.05

Sec-O-glucosylhamaudol								
0.004	2.21	−2.17	4.21	−4.39	5.24	2.85	3.63	−5.34
0.041	4.13	−1.18	3.83	2.09	4.82	−3.53	5.96	−4.77
0.414	3.90	−1.71	2.26	1.06	3.70	−1.85	4.73	−2.19

**Table 5:** Pharmacokinetic parameters in normal and febrile rats after oral administration of Radix *Saposhnikoviae* (RS) extract (mean ± SD, *n*=6).

Group	Analytes	*T*_1/2_ (min)	*T*_max_ (min)	*C*_max_ (*μ*g/mL)	AUC_0–*t*_ (*μ*g/mL ∗ min)	AUC_0–∞_ (*μ*g/mL ∗ min)
Normal	Prim-O-glucosylcimifugin	2.84 ± 0.85	0.50 ± 0.00	0.36 ± 0.07	0.54 ± 0.07	0.56 ± 0.08
	Cimifugin	8.08 ± 4.59	6.00 ± 1.27	1.86 ± 0.18	27.50 ± 1.99	32.61 ± 6.41
	4′-O-*β*-D-glucosyl-5-O-methylvisammino	1.67 ± 0.38	0.47 ± 0.07	0.11 ± 0.01	0.17 ± 0.04	0.18 ± 0.05
	5-O-methylvisammino	6.35 ± 2.87	8.33 ± 0.82	0.38 ± 0.09	4.47 ± 0.99	5.08 ± 0.74
	Sec-O-glucosylhamaudol	20.54 ± 16.51	0.42 ± 0.14	0.05 ± 0.01	0.13 ± 0.02	0.15 ± 0.04

Model	Prim-O-glucosylcimifugin	11.59 ± 7.49	0.31 ± 0.07^*∗*^	0.46 ± 0.06^*∗*^	0.66 ± 0.12	0.67 ± 0.12
	Cimifugin	7.79 ± 2.92	2.17 ± 0.41^*∗*^	2.29 ± 0.18^*∗*^	26.85 ± 1.41	32.03 ± 4.67
	4′-O-*β*-D-glucosyl-5-O-methylvisammino	7.41 ± 6.60	0.33 ± 0.00^*∗*^	0.15 ± 0.01^*∗*^	0.23 ± 0.02	0.24 ± 0.03
	5-O-methylvisammino	6.21 ± 1.20	8.67 ± 1.03	0.40 ± 0.05	5.04 ± 0.38	5.68 ± 0.58
	Sec-O-glucosylhamaudol	6.99 ± 3.86	0.36 ± 0.07	0.05 ± 0.01	0.13 ± 0.01	0.14 ± 0.01

## Data Availability

The data used to support the findings of this study have been deposited in the Figshare repository (10.6084/M9.figshare.7375871).
